# Prerequisites for providing effective support to family caregivers within the primary care setting – results of a study series in Germany

**DOI:** 10.1186/s12875-021-01601-x

**Published:** 2021-12-22

**Authors:** Julian Wangler, Michael Jansky

**Affiliations:** Center for General Medicine and Geriatrics, University Medical Center of the Johannes Gutenberg University Mainz, Am Pulverturm 13, 55131 Mainz, Germany

**Keywords:** Caregivers, General practitioner, Ientification, Strain, Needs, Care, Support

## Abstract

**Background:**

General Practitioners are considered to be well placed to monitor home-care settings and to respond specifically to family caregivers. To do this, they must be sensitive to the needs and expectations of caregivers. In order to determine the current status of GP care in terms of the support given to family caregivers, a series of studies were conducted to gather the perspectives of both caregivers and GPs. The results are used to derive starting points as to which measures would be sensible and useful to strengthen support offered to family caregivers in the primary care setting.

**Methods:**

Between 2020 and 2021, three sub-studies were conducted: a) an online survey of 612 family caregivers; b) qualitative interviews with 37 family caregivers; c) an online survey of 3556 GPs.

**Results:**

Family caregivers see GPs as a highly skilled and trustworthy central point of contact; there are many different reasons for consulting them on the subject of care. In the perception of caregivers, particular weaknesses in GP support are the absence of signposting to advisory and assistance services and, in many cases, the failure to involve family caregivers in good time. At the same time, GPs do not always have sufficient attention to the physical and psychological needs of family caregivers. The doctors interviewed consider the GP practice to be well suited to being a primary point of contact for caregivers, but recognise that various challenges exist. These relate, among other things, to the timely organisation of appropriate respite services, targeted referral to support services or the early identification of informal caregivers.

**Conclusions:**

GP practices can play a central role in supporting family caregivers. Caregivers should be approached by the practice team at an early stage and consistently signposted to help and support services. In order to support care settings successfully, it is important to consider the triadic constellation of needs, wishes and stresses of both the caregiver and the care recipient. More training and greater involvement of practice staff in the support and identification of caregivers seems advisable.

## Background

In the EU-27, over 20% of the population is already over 65 years old [1, 2]. This results in a growing need for care and support. In Germany, this need is documented on the basis of approx. 4.1 million people formally classed as needing care [3]. If informal unremunerated care and support activities are also considered, this number increases to approximately 5.5 million who receive care or support [4, 5].

Informal care is predominantly provided in the home environment by private citizens, who bear a considerable share of the caregiving burden in caring for people close to them who are in need of care [6–8]. According to representative data, more than 17% of 40- to 85-year-olds regularly support at least one person in coping with everyday life; of these, a good third provide care in the stricter sense [9, 10].

Although research has shown that a caring role can provide a subjective sense of purpose [11, 12], it is associated with a greater health risk due to the physical and mental strain involved [8, 10, 13–19]. If the consequences of the illness have not been considered in advance and precautionary measures have not been taken, it is not uncommon for caregivers to become burnt-out and exhausted [15, 20–22]. In order to avoid such crises and to promote the resilience of caregivers, various support services have been established in Germany, including care support centres, outpatient psychiatric services and dementia networks [23]. However, studies show that such services are only used by a proportion of caregivers [24–26].

Since they have provided ongoing care to the patient over many years and know them well, GPs are considered to be well suited to provide support for home care settings and to respond to the particular concerns of family caregivers [6, 27–29]. Apart from diagnosing and treating health problems, GPs can provide information and advice to caregivers, offer psychosocial support and gain an overall picture of the care conditions so that needs can be addressed promptly. By referring patients to support and counselling services, GPs can set the course for successful long-term care and show caregivers ways to offset and relieve the burden of caregiving [24, 30].

In 2018, the National Association of Statutory Health Insurance Physicians (KBV) carried out a telephone survey of 6043 randomly selected citizens representative of the German resident population. The study concluded that about 60% of family caregivers talk to their GP about their caring role [29]. Of these, around two-thirds had been made aware of concrete offers of help by their GP. Up to now, there has been a lack of reliable studies, especially for the German-speaking countries, which shed light on the status of GP support for the target group of family caregivers, but also on the practical challenges experienced, both broadly and from multiple perspectives, i.e. from the point of view of doctors and caregivers.

## Methods

### Overall study and research interest

This paper wants to help determine the current status of German GP care in terms of the support given to family caregivers. By doing so, it summarises the results of a series of explorative studies conducted to gather the perspectives of both family caregivers and GPs, and compares the results with existing research.

The study, which consists of three sub-studies, stands as an independent supplementary study in the broader context of an Innovation Fund model project on outpatient medical and nursing dementia care *(DemStepCare*) [31]. All three sub-studies have already been published or accepted for publication. The specific purpose of the present work was to bundle commonalities of the individual studies from an overarching perspective and to draw conclusions in terms of an overall view of the study series. We are convinced that interconnecting the three studies in this way opens up a concentrated view and increases the informative value of all studies.

The aim was to explore the attitudes, experiences and wishes of caregivers and GPs with regard to the support of caregivers provided by the GP setting. The focus was on the importance of GP support for caregivers and how GPs perceive their own remit as contact partners. One focus was to compare the needs of caregivers with the support they actually experience. Another aim was to identify the challenges for GP care.

Against the backdrop of the joint consideration of all central results, the article aims to derive starting points as to which measures would be sensible and useful to strengthen support offered to family caregivers in the primary care setting. In view of this focus, special attention is paid to weaknesses in the GP setting.

### Sub-studies

Initially, an online survey of 612 family caregivers [32] was conducted in spring 2020 to identify care needs and experiences in relation to GP care. The anonymous survey was posted on 17 German-language Internet forums aimed at family care and family caregivers. The selected forums were usually embedded in general information portals on the subject of care. These websites are intended to support family caregivers across the board on a wide variety of questions relating to care in a domestic setting (no specific clinical pictures) and enable an exchange. Based on the registered number of members, the authors assume that the forums theoretically reach up to 11,000 family caregivers in total. In order to obtain the broadest possible picture of the reality of care in Germany, the inclusion criterion was deliberately kept general; accordingly, the survey target group included all kinds of family caregivers. The mean age of the respondents was 54 years, with 93% of the respondents being women.

In a next step, we wanted to explore these results in more detail, a total of 37 family caregivers were recruited from the same online care forums and interviewed between autumn 2020 and spring 2021 [33]. In the respective Internet forums, a call was made in the form of a thread in which information was given on the general topic. People who were willing to be available for an interview could contact the given email address. As in the online survey, the interviews with family caregivers essentially included all types of caregivers and care constellations. The inclusion criterion was that the caregivers had regularly cared for at least one relative, friend or neighbour in the last 12 months.

In a next step, the attitudes and experiences of GPs with regard to the care of caregivers were gathered by means of an online survey [34]. In spring 2021, all 13,170 GPs in Baden-Württemberg, Hesse and Rhineland-Palatinate were invited to participate in the anonymised survey by post. In the one-time letter of invitation, the doctors were given, among other things, password-protected access to the online survey. Of the 3595 questionnaires processed, 3556 fully completed questionnaires were included in the evaluation (response rate: 27%). This survey determined, inter alia, the priorities set by GPs when supporting caregivers and to what extent they use the available resources to make care more effective. The mean age of the GPs surveyed was 55 years, with about half of the doctors having their practice in rural regions.

Incentives were not used in any of the three studies.

### Development of survey instruments

Since the studies built on each other, there was a continuous learning process with regard to the design of the subsequent sub-study. In addition, the survey instruments developed were supported by other elements:

- Preparations for the multi-part study series (including interviews with family caregivers in the context of *DemStepCare*, focus group with 8 GPs)
- Further preliminary studies by the authors on dementia care by GPs (e.g., [35])
- General literature search (papers used here focused on caregivers and their support in the GP-based setting [12, 17, 24, 28, 30, 36, 37], including those by Geschke et al. [24], Greenwood et al. [28, 36] and Joling et al. [17].
- Carrying out pre-tests in the run-up to data collection

The aim was to keep the instruments used to interview family caregivers and GPs mutually compatible. For this purpose, certain question models were adapted to facilitate comparison of the results.

### Data analysis

Data from the quantitative studies were evaluated using SPSS 23.0. Apart from the descriptive analysis, a T-test was applied to independent random samples in order to identify significant differences between two groups. In the case of the survey of family caregivers, binary logistic regression was used to identify possible influencing variables. Evaluation of the interview study as well as the open questions in the questionnaires is based on a qualitative content analysis [38].

## Results

**Fig. 1:**
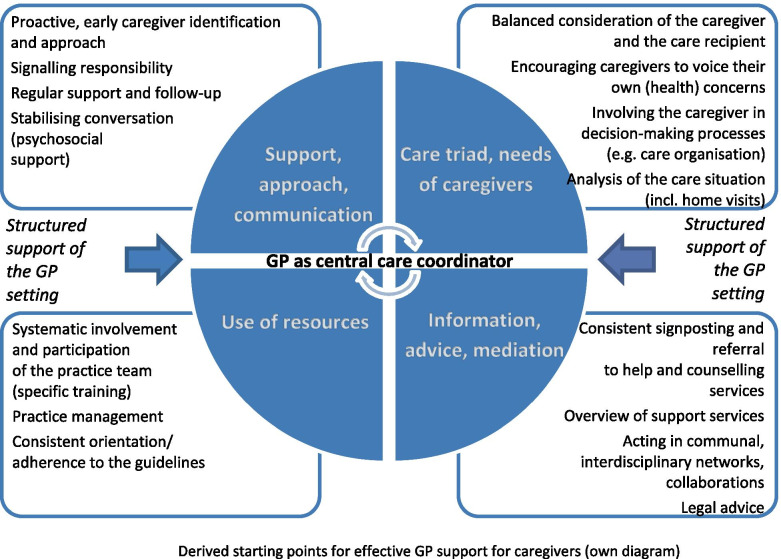
Derived starting points for effective GP support for caregivers (own diagram)

Figure 1 shows the starting points condensed from the analysis of the sub-studies with a view to more effective GP support for family caregivers. In the following, each of the dimensions presented will be discussed with reference to the respective central findings and correlated with the existing research.

### Support, approach, communication

As shown in the survey of family caregivers [32], caregivers experience GPs as highly skilled and trustworthy central points of contact. Three out of four respondents (72%) talk to their GP about their caring role, with 54% doing so frequently. The way in which support is provided is judged positively in important contexts, especially the GP’s knowledge of the personal care situation, approachability on a wide range of problems and the attention given to the person in need of care.

At the same time, the same survey shows that the wishes of caregivers with regard to an early approach by the GP practice are frequently not matched by their own experience. Fewer than one in two caregivers (42%) report having been promptly identified as such by their GP. One in five (18%) reports that the responsible GP did not wait for them to voice questions or problems regarding care but (pro-)actively approached the caregiver. In the qualitative interviews [33], some of the caregivers stated that they had initially felt uncertain about the extent to which their needs and problems should be a matter for GP support; there was hesitance, which sometimes deferred problematic situations. Accordingly, counselling sessions in the initial or preparatory phase of care are comparatively less frequent.

Such findings from the interviews with family caregivers are in line with the survey of GPs [34], which showed that the latter perceive it as a great challenge to systematically identify informal caregivers in their daily practice (59%). As other papers have pointed out, transitions to becoming informal caregivers are often fluid, so it can be difficult for the GP team to identify them [7, 27, 28, 36, 39]. Difficulties arise especially if the person receiving care is not registered with the same practice as the caregiver [10]. Krug et al. [40] note that identification of relatives and their problems is often more likely to be in response to stress behaviours identified by the practice team. Because of this, caregivers are often not registered until stress or even decompensation processes are well advanced. In this respect, it is extremely important that GP responsibility for caregivers is explicitly signalled [37, 41, 42].

As the results of the sub-studies have shown, there is a comparatively large variation in the regularity and thus the interval between GP support consultations. In surveys and interviews [32, 33], caregivers take issue with a not infrequently rushed, irregular and sometimes rather casual treatment of their caring situation, which it is often taken up by other reasons for consultation (e.g., health check-ups, vaccinations).

GPs often cite time constraints as a significant challenge to providing adequate advice to caregivers in everyday practice (68%); in addition, many GPs find it challenging to ensure a regular exchange with caregivers (43%), e.g. because the caregiver has a different GP. Linked with such barriers to support is the fact that GPs are often unable to adequately meet the need expressed by caregivers for a stabilising, psychosocial discussion [32, 33].

### Care triad and needs of caregivers

As already mentioned, the study results from both perspectives reflect that GPs see themselves as contacts who are well acquainted with the situation of family caregivers and have a generally accurate picture of their personal situation. Family caregivers are remarkably positive about the way in which GPs create insight on the part of the care recipient by offering explanations (85%) and involve them in decisions (82%). In contrast, slightly more than half of the caregivers interviewed report feeling that their views, needs and stresses were adequately considered by the GP; 23% feel encouraged to address their own health situation [32].

The latter finding should be regarded with caution, since informal caregivers often trivialise their own complaints compared to the extent of the problems of the person cared for and consider potential complaints to be relative [39, 43]. Nevertheless, the results of the other studies also confirm that caregivers and their concerns generally receive far less consideration, given the time and resource constraints for GPs. In the interviews with caregivers [33], it was expressed that GPs are often mainly focused on the person being cared for, without considering the needs and stresses of the caregiver. On the other hand, 44% of GPs report that they find it challenging to consider the needs and wishes of both the caregiver and care recipient in their daily practice [34].

The research literature confirms such findings. Due to the often tardy and inconsistent identification of caregivers and the somewhat sporadic contacts with them, GPs find it difficult to involve caregivers from the outset [32, 37, 38]. On the other hand, in the triadic constellation there is a tendency for GPs to perceive caregivers primarily in terms of their function relative to the person being cared for, so that psychosocial effects are marginalised [27, 36, 39, 43].

Against this background, the GP team should empathically encourage caregivers to voice their own health concerns and offer support (consultations independently of the care recipient, where appropriate), as well as refer them to specific support services [37, 41, 42]. It is also important to involve caregivers in decision-making processes about adaptation of the care (organisation) [6, 15, 17]. Home visits can also help to better assess care and stress situations. The survey of family caregivers [32] has shown that it is not yet possible to adequately fulfil caregivers’ wish to be visited by the GP team in their private premises.

### Information, advice, mediation

In general, caregivers positively rate the information and advisory activities of GPs with regard to specific clinical pictures and courses of disease, as well as diagnostic and treatment options. One weakness identified in all the sub-studies is that GPs do not always provide referrals to counselling or support services. In the survey of family caregivers [32], for example, 60% report having been referred to support and care services by their GP at least once. The results of a regression analysis show that referrals to such services are an important factor influencing the feeling of being able to cope with the care situation. The interviews [33] also showed that a considerable proportion of caregivers would like the GP to play a greater advisory role when it comes to organising the framework conditions for care.

Among the GPs surveyed [34], more than three quarters (79%) found it very challenging to point caregivers to the appropriate support and respite services in the area. 48% of doctors interviewed believe that they have made at least half of the family caregivers they have supported aware of concrete offers of help in recent years, day-care facilities or short-term care and care services being mentioned in particular. In response to open questions, doctors with rural practices in particular cite the lack of interprofessional structures (e.g. bridging care services, inpatient palliative care facilities) and bureaucratic hurdles as the cause for limited mediation.

In general, these results correspond with the finding in the research literature that GPs often do not have an adequate overview of external forms of support for caregivers [24, 30] and are mostly not integrated into community health networks or (in)formal collaborative networks [43–46]. Another frequently encountered problem is the lack of availability of certain forms of assistance at short notice. For example, the GP survey [34] showed that 89% of all doctors experience the rapid availability of care or psychosocial respite services as a challenge.

### Use of resources

In the course of the overall study, we were able to identify several practical resources which, if their use was mandatory, can contribute to more effective support for family caregivers. One of these is the involvement of practice staff. Particularly when it comes to the identification of informal caregivers, it is important that this should not be seen as the exclusive task of GPs but, if it is to be effective, as a task for the entire practice team [37, 42, 47]. Accordingly, it is important to make non-clinical practice staff (e.g. non-clinical practice assistants, primary care assistants) aware of the need to identify caregivers.

Assuming that they genuinely receive appropriate training and are involved in the support of caregivers, non-clinical practice staff can also offer potential synergies when it comes to carrying out home visits. Even the assumption of advisory and coordinating roles (e.g. referral to local support services) can relieve the burden on GPs and at the same time strengthen the mediator role of the GP practice [40]. Last but not least, practice staff can offer a lot of added value when it comes to providing (initial) psychosocial support and, if necessary, linking this to referrals to stabilisation services.

Practice management is particularly important when it comes to involving practice staff. Firstly, this is about creating the conditions that allow caregivers to be readily observed (e.g. rotation of staff between different duties) [37]. Secondly, obligatory and systematic arrangements are required for documenting specific problems (e.g. references in the patient notes about caregiving role or signs of stress) [40, 47].

One problem is that, so far, GPs have only partially involved their practice staff in the identification and support of caregivers. For example, 47% of the GPs surveyed [34] reported having members of their non-clinical practice team who regularly support their own work in terms of identifying and supporting family caregivers. Similarly, only some GP practice staff are trained to undertake specific tasks associated with this. Similar findings have already been identified in several studies on the diagnosis of dementia in the primary care setting, where the general practice team has so far only been involved to a limited extent in observing elderly patients and looking out for and/or documenting warning signs [35, 47].

Beyond the practice staff, another significant resource is the application of and compliance with evidence-based guidelines. The S3 guideline “Family caregivers” was published in Germany for GPs as early as 2005 and has since been updated and expanded [41]. With regard to the above-mentioned DEGAM guideline, 40% of the GPs surveyed report that they are aware of it. Of these, 55% reported using the guideline frequently or occasionally (44% rarely). Such results are consistent with other reports of the critical distancing of some GPs from guidelines published by medical societies in particular [48–50].

### Status quo and starting points for optimisation

Overall, 68% of the caregivers surveyed who talk to their GP about care say they feel (very) well supported by the GP. 70% feel that their GP is usually good at helping them when they approach them with a care-related question.

47% of the GPs surveyed stated that there was a (very) good possibility of meeting the needs of family caregivers in their everyday practice (53% less good or not good at all). The possibilities and structures that exist for GPs within the healthcare system to provide good support for caregivers are assessed positively by 44%, and rather negatively by 52%.

In terms of an overall assessment, it appears that the vast majority of doctors (77%) consider the GP setting as the primary contact point for the needs of caregivers. However, many respondents (56%) say that, when it comes to playing a more proactive role for this target group, they are limited by the current framework conditions.

In response to an open question, some of the doctors said that, in order to be able to better support caregivers in the future, they wanted to see better integration of GPs within local health and care structures or a closer collaboration in the interprofessional network, so as to give them a better overview of existing services and the ability to make targeted referrals. In addition, they express the wish for the health insurance funds to systematically support family caregivers, thereby assisting the work of GPs. Another suggestion is the creation of a low-threshold support programme, in which caregivers can be enrolled by GPs and which, on the basis of an individual risk stratification, guarantees them ongoing information and advice, as well as intervention measures when needed.

## Discussion

### Principal findings and comparison with prior work

The study series was able to generate a broad picture of the current status of GP care with regard to support for family caregivers. Due to their position in the German health care system, GPs perform extensive primary care tasks. GPs are the first point of contact for patients and therefore often familiar with their patients and the patients’ family members for many years; there is a trusting doctor-patient relationship [6, 27–29].

The results obtained in the course of the sub-studies show that the GP setting has great potential to act as a central support for this group. Discussions with family caregivers about care (organisation) and care circumstances are widespread in everyday practice and are based on a high level of trust on the part of caregivers. Especially the low-threshold accessibility for various problems, the familiarity with the personal circumstances as well as the attention to the person in need of care are experienced positively.

This confirms previous studies which underline the major importance of GP support for the target group under consideration and see GPs as being in a position to make key contributions to the longer-term stabilisation of home-care settings [6, 7, 14, 28–30, 51, 52]. Both caregivers and GPs believe that the primary care setting has great potential to address and deal with the problems of caregivers [7, 14, 29, 30, 52]. For example, a study conducted in Ireland highlights the priority role of the GP in developing longer-term coping and resilience strategies in home-care settings [53]. For their part, Greenwood and colleagues [30] were able to work out that the primary care setting can play a central role in supporting and relieving the burden on caregivers and effectively coordinate further care.

Nevertheless, the results of the present study also reveal weaknesses which mean that, despite being very aware of the need to support family caregivers, GPs are not always able to meet the needs of home-care situations as part of their everyday practice [6, 51, 54]. This is true, for example, with regard to the role of GPs in identifying and anticipating care difficulties. Caregivers would also like the GP to play a greater advisory role when it comes to organising the framework conditions for care and signposting them to help and support services. Additionally, the sub-studies confirmed the findings from previous studies that GPs do not always consider the physical and emotional needs of family caregivers to the same extent as those of the person requiring care [30, 36, 37, 39, 42, 52].

In particular, the comparatively low level of GP referral activities and collaboration with support services in the provision of care results in restrictions and delays in the effective support and (preventive) stabilisation of caregivers. As noted in various studies, GPs in Germany - especially in rural regions - are often solitary providers and cannot access interprofessional networks and collaborations [24, 26, 30, 40, 43–46, 54]. The results of the survey of family caregivers are confirmed, for example, by a Canadian study conducted by Parmar et al., who find that GPs fail to consistently address the need of caregivers and care recipients for early and regular signposting to respite services [45, 55]. When family caregivers are referred to such support services, they benefit from timely access to information on organising care [8, 52], which allows the caregiver to stay at home longer without care crises (e.g., hospitalisations) arising [24, 56].

Another issue is that the GP team does not always identify family caregivers in a timely and systematic way, making it harder to identify specific needs and anticipate pressures. Overall, the results demonstrate the value of active communication by the GP team in relation to the family caregiver group. In the qualitative studies by Burridge et al. conducted in Australia, it is notable that caregivers do not always feel confident to voice their problems, if GPs do not signal to them that they see themselves as a point of contact [39, 57]. Against this backdrop, it makes sense to strengthen GPs’ conversation skills in dealing with caring relatives through further training. If communication can be more open between both parties, family caregivers will be less reluctant to report feelings of burden, depression, and stress [51]. A systematic assessment of the caregivers’ general well-being, performed by the GP, is essential for the prompt adjustment of home care [58].

A fundamental problem not only of the German, but also of other health systems is fragmentation, meaning that the sectors are separated. As a result, primary care is often not integrated into multi-professional care, which also affects the care of family carergivers [59]. In Germany in particular, there is often a lack of staff who can relieve and supplement the GP, offer support to caregivers and competently assign them to support services [30].

In this context, it is worth mentioning that only a proportion of GPs train non-clinical practice staff and involve them so that they can take on specific tasks such as identifying and supporting family caregivers [24, 30, 40, 47]. Studies like those by Krug et al. [40] show that the detection of exhaustion in caregivers is not systematic among staff members, but rather a reaction to warning signals that the caregivers show to the practice team. This problem is often related to a lack of knowledge and awareness [32, 35]. At the same time, various studies show that there is a great need for delegation in primary care since GPs are often overworked already in most countries [47]. Therefore, practice staff should be more systematically involved in the detection and support of family caregivers [35]. Staff members who have undergone appropriate training can also take on referring and mediating activities to advisory and support networks. If the practice team is networked with other service providers, this not only relieves caregivers, but also the practices themselves; the mediator role of the GP’s practice can be strengthened. Requests made to the practice team could then be passed on to competent actors in the network. For example, closer cooperation with long-term care insurance funds, which GPs sometimes use in the context of care advice [40], and the local care support points could help relieve caregivers. Where such collaborative solutions exist in everyday practice, GPs also find it much easier to meet the needs of caregivers [51]. Practice management is of particular importance with regard to the involvement of the practice staff. On the one hand, prerequisites should be created under which it is possible to identify and observe caregivers (e.g. regularly changing work areas). On the other hand, it depends on systematic arrangements with regard to the documentation of abnormalities (e.g. entering signs of stress in the patient file) [37, 42].

In order to stabilize home care settings, there is also the need for structured interdisciplinary forms of care that combine medical, nursing and further care offers in order to offer person-centered and evidence-based support [60–62]. The lack of effective outpatient crisis intervention structures often leads to hospital admissions in crisis situations, which may result in serious complications for patients [63]. There is some discussion on the introduction of case and support managers to assist GPs in supporting family care situations [52, 59, 64, 65]. Case managers offer the advantage that they are cross-sectorally networked and can act as a link between GPs and other care providers (e.g. care services, support networks, emergency clinics), so that risk stratifications for those in need of care and carergivers can be carried out at an early stage [59].

Also important in care planning is the issue of adequate referral to care-supporting systems, networks and services. In this context, however, it has been found that GP teams often complain about inadequate integration into professional care and advisory networks [40]. A central lever for making GP support for family caregivers more effective is undoubtedly the closer integration of GPs into counselling and support services [66]. To this end, it will be important to strengthen interdisciplinary communication, to establish collaborative municipal networks in the area of health promotion [44, 58] and to provide GPs with a reliable knowledge of advisory services in their area in order to facilitate the straightforward referral of caregivers. A systematic review by Plöthner et al. points out the importance of strengthening outpatient care structures [51]. The researchers draw the conclusion that establishing an outpatient care system, which supports families and friends in providing (elderly) care while meeting the needs and wishes of informal caregivers, is of high relevance. An important prerequisite for this is to take into account family doctors with their own contractual elements, ensuring that they are appropriately remunerated when they take on advisory, mediating and caring activities for a caregiver network [21, 51, 56]. Scientifically supported model projects are already trying to strengthen the anchoring of GP-based care in regional advisory and support networks [30, 31, 59, 60].

The increased focus on evidence-based guidelines is also an important tool for better addressing the needs of caregivers. For example, manageable care plans derived from guidelines could help GPs tailor care management to the care needs of the caregiver and the patient [46, 49]. In doing so, the assessment of the care situation and its impact on the general well-being of the caregiver can approached in a structured way [66]. Clear and efficient guidelines from early diagnosis to adequate referrals can certainly improve the GP’s ability to support time- and energy-consuming home-care situations. Consequently, intervention trials focusing on the skills of GPs could be helpful in improving home-care outcomes regarding the family caregiver [32, 37].

Not only in Germany, but also internationally, there is a lack of longitudinal studies that include doctors, nurses (e.g. palliative care patients) and family caregivers in order to support the development and effectiveness of family GP-related interventions [67] that maintain or increase the quality of life of patients and their relatives [68]. An exception is the implementation of the Gold Standards Framework in Great Britain, in which family caregivers are explicitly included [69]. The caregivers‘perspectives and experiences were taken into account, e.g. the need for a professional coordinator [70] and the support of district nurses [71]. The extent to which such approaches can be adopted in the more fragmented German health system is part of future research projects.

### Starting points

The following starting points for effective GP support for family caregivers can be stated against the background of the findings as well as the results from previous studies:

- Early identification, approach and involvement of (informal) caregivers is essential for providing good support [72]. For example, possible care activities can be consistently queried using anamnesis questionnaire when new patients have initial contact with the practice. In addition, it seems worthwhile to design postings in the reception area of the practice and give advice from the practice team that family caregivers should identify themselves (if necessary, design in several languages). People in need of care should be asked who their informal caregivers are. In the case of new diagnoses that are known to be associated with a need for care, the practice team should ask about possible caregivers. Information with regard to care constellations can also be requested during home visits as well as informal caregivers can be identified. Patients with a presumed role as caregivers should be addressed about the issue [37, 42]. Moreover, it would be beneficial if caregiving relatives also voluntarily point out their care activities and speak to GPs about this issue. This also requires health policy activities that emphasise how important it is for family caregivers to approach GPs on their own initiative and build a stable relationship [41, 42].
- In the context of early identification and crisis prevention, non-clinical practice staff could be more closely involved, and tasks could be delegated by the GP. To this end, GPs should invest more in special further training and in optimised practice management. So far, practice teams do not systematically record signs of stress and exhaustion in caring relatives. An entry in the patient file stating whether someone is a caring relative or which family member is mainly responsible for the care could provide a remedy here and provide an initial indication of whom to pay particular attention to in terms of excessive demands from a care situation. The same applies to the observation and documentation of warning signals. Caregiving relatives could be proactively identified by the practice team during initial contact and during house calls, but also by actively asking the person in need of care [37, 51, 55, 65]. For internal communication and observation in the practice, a catalog of questions can be developed on perceptions with regard to contact with family caregivers [41]. The systematic recording of burdens and resources of caring relatives and the networking with other service providers as well as the knowledge of their offers facilitate appropriate intervention.
- Family caregivers should be made aware that their support falls within the remit of the GP practice, so that any health concerns are articulated without delay. Similarly, it seems advisable not to wait for caregivers to raise problems but rather to proactively take the initiative (e.g. via opportunities such as health checks or vaccinations). Caregivers should be empathically encouraged to raise their own health concerns [37].
- GP practice teams should be made more aware that, within the triadic constellation, the needs, wishes and pressures of caregivers are key to the success of longer-term care [27, 32, 36, 39, 43]. If necessary, consultations should be arranged independently of the person being cared for and sufficient time should be made available, e.g. during a home visit [27, 46].
- The potential of practice staff can be used through targeted training, not only in identifying caregivers but also in advising caregivers and making home visits to better address care problems. In this context, psychosocial skills could be also expanded through further training. Such additional tasks taken over by members of the practice staff should be given greater consideration in the remuneration of the practice team [47, 65].
- It seems advisable to raise GPs awareness of the existence and benefit of evidence-based guidelines, especially with regard to supporting family caregivers [42, 72]. The Burden Scale for Family Caregivers (BSFC-s) should be used for the standardized recording of burdens [41].
- Family caregivers should be consistently involved in decisions with respect to the organisation of care right from the start. Caregivers too often feel bypassed when it comes to support of home care. In addition, studies reveal that interventions that are not previously discussed with the caregiver and which occur in an acute situation fail to achieve the expected result [66].
- Consistent and early mediation to concrete help and support services gives family caregivers timely access to information about the organisation of care; the risk of caregiver ‘burnout’ is significantly minimised [8, 10, 21, 24, 30, 45]. If family caregivers are suitably monitored, outpatient care can be arranged so that caregivers can stay at home longer [56, 64].
- The structural support for primary care as well as the intersectoral connection of GPs’ practices should be strengthened. In the role of multi-professional actors, case managers can mediate between GPs, patients and caregivers as well as other offers of help and, thereby, overcome the limits of a fragmented health system [52, 59, 61, 64]. A central lever for making GP support for family caregivers more effective is undoubtedly the closer integration of GPs into counselling and support services. To this end, it will be important to strengthen interdisciplinary communication, to establish collaborative networks in the area of health promotion [44, 58] and to provide GPs with a reliable knowledge of advisory services in their proximity in order to facilitate the straightforward referral of specific caregivers (e.g. Parkinsonism, Stroke, Dementia). A good knowledge of local conditions and effective networking of the practice team with other professional providers contribute to improved care for caregivers while strengthening their information and education as well as the prevention of care crises [21, 51, 56]. To this end, help and support services need to be systematised so that GPs have an overview and consultations can be structured and still be tailored to the individual needs of those affected. For some family caregivers, advice and written information will be sufficient; others will need more support and guidance. It could be worthwhile for GPs to take initiatives to improve their formal and informal cooperation with counseling and support actors in the field of community care. In that regard, e.g. doctor or practice networks offer great opportunities. However, this is primarily a task for structured municipal health promotion [44]. The establishment of health and prevention networks is associated with considerable advantages.

### Strengths and limitations

This paper has helped in a comprehensive and multi-methodical way to identify information on the status quo of caregiver support in primary care. Because of the broad, heterogeneous and widely dispersed samples the results have national significance.

However, the sub-studies fail to provide a representative picture of opinion, due to the limited number of cases and the self-selection of respondents, since the surveys were conducted online. One has to allow for the fact that older people are less au fait with technology, so that older caregivers and GPs might be under-represented in the sample. Accordingly, it can be assumed that the recruitment of caregivers in other settings (e.g. waiting room surveys in GP offices) would lead to potentially more generalisable statements about the population under consideration. Such studies should be conducted with a view to optimising primary care with regard to the needs of family caregivers.

It should also be borne in mind that caregivers were deliberately considered very broadly and that the specific needs of different subgroups (e.g., those caring for dementia patients) could therefore not be considered separately.

## Conclusions

GPs are very important to family caregivers for providing information on planning and organising care, as well as psychosocial support and reassurance. By responding to the needs of caregivers, GPs are able to stabilise home-care settings in the longer term and avert care crises.

The results show that family caregivers see GPs as a highly skilled and trustworthy central point of contact. In the perception of caregivers, particular weaknesses in GP support are the absence of signposting to advisory and assistance services and, in many cases, the failure to involve family caregivers in good time. At the same time, GPs do not always have sufficient regard for the physical and psychological needs of caregivers. The doctors interviewed consider the GP practice to be well suited to being a primary point of contact for caregivers, but recognise that various challenges exist. These relate, among other things, to the timely organisation of appropriate respite services, mediation to appropriate assistance or the early identification of informal caregivers.

Ideally, family caregivers – provided that the GP team is aware of their care activities – should be approached by the practice team at an early stage and consistently signposted to help and support services. To this end, it will be important to strengthen interdisciplinary communication, to establish collaborative (municipal) networks in the area of health promotion and to provide GPs with a reliable knowledge of advisory services in their proximity. In order to support care successfully, it is important to consider the triadic constellation of needs, wishes and stresses of both the caregiver and the care recipient. More training and greater involvement of practice staff in the support and identification of caregivers seems advisable.

## Data Availability

All major data generated or analysed during this study are included in this published article. Additional information can be provided on request.

## Abbreviation

GP(s): General Practitioner(s)

## References

[1] Eurostat. Population structure and aging. 2021. Available from: URL: https://ec.europa.eu/eurostat/statistics-explained/index.php?title=Population_structure_and_ageing. [Cited 2021 Sep 15].

[2] WHO Regional Office for Europe (2015). Home Care in Europe.

[3] Statistisches Bundesamt [Federal Office of Statistics]. Pflegestatistik 2019 [Care statistics 2019]. Available from: URL: https://www.destatis.de/DE/Themen/Gesellschaft-Umwelt/Gesundheit/Pflege/Publikationen/_publikationen-innen-pflegestatistik-deutschland-ergebnisse.html. [Cited 2021 Sep 15].

[4] Nowossadeck S, Engstler H, Klaus D: Pflege und Unterstützung durch Angehörige [Care and support by family members]. Report Altersdaten 1/2016. Berlin: Deutsches Zentrum für Altersfragen [German Centre of Gerontology]; 2016.

[5] Klaus D, Tesch-Römer C, Mahne K, Wolff J, Simonson J (2016). Pflege und Unterstützung bei gesundheitlichen Einschränkungen: Welchen Beitrag leisten Personen in der zweiten Lebenshälfte für andere? [Care and support in health disabilities: What contribution do people in the second half of life give to others?]. Altern im Wandel: Zwei Jahrzehnte Deutscher Alterssurvey [Ageing in transition: The German Ageing Survey two decades on].

[6] Connell CM, Boise L, Stuckey JC (2004). Attitudes toward the diagnosis and disclosure of dementia among family caregivers and primary care physicians. Gerontologist.

[7] DAK: DAK-Pflege-Report 2015 [DAK care report 2015]. Hamburg: DAK-Gesundheit; 2015.

[8] Wuttke-Linnemann A, Henrici CB, Müller N, Lieb K, Fellgiebel A (2020). Bouncing back from the burden of dementia: predictors of resilience from the perspective of the patient, the spousal caregiver, and the dyad — an exploratory study. GeroPsych.

[9] Linnemann A, Hilsenbek MM, Lelieveld I, Geschke K, Wolf D, Fellgiebel A (2020). Comparison of psychosocial and medical characteristics of patients with dementia and their primary informal caregivers between inpatient and day clinic treatment. Dementia.

[10] Schmidt M, Schneekloth U (2011). Abschlussbericht zur Studie, Wirkungen des Pflege-Weiterentwicklungsgesetzes [Final report from the study on the effects of the law concerning further development of care].

[11] Bestmann B, Wüstholz E, Verheyen F (2014). Belastung und sozialer Zusammenhalt. Eine Befragung zur Situation von pflegenden Angehörigen. WINEGWissen 04.

[12] O’Reilly D, Connolly S, Rosato M, Patterson C (2008). Is caring associated with an increased risk of mortality? A longitudinal study. Soc Sci Med.

[13] Beach SR, Schulz R, Williamson GM, Miller LS, Weiner MF, Lance CE (2005). Risk factors for potentially harmful informal caregiver behavior. J Am Geriatr Soc.

[14] Cherry MG, Salmon P, Dickson JM, Powell D, Sikdar S, Ablett J (2013). Factors influencing the resilience of carers of individuals with dementia. Rev Clin Gerontol.

[15] Schulz R, Sherwood P (2008). Physical and mental health effects of family caregiving. Am J Nurs.

[16] Dias R, Santos RL, Sousa MF (2015). Resilience of caregivers of people with dementia: a systematic review of biological and psychosocial determinants. Trends Psychiatry Psychother.

[17] Joling K, Windle G, Dröes R-M (2016). Factors of resilience in informal caregivers of people with dementia from integrative international data analysis. Dement Geriatr Cogn Disord.

[18] Roepke SK, Mausbach BT, Patterson TL (2011). Effects of Alzheimer caregiving on allostatic load. J Health Psychol.

[19] Laporte Uribe F, Heinrich S, Wolf-Ostermann K (2017). Caregiver burden assessed in dementia care networks in Germany: findings from the DemNet-D study baseline. Aging Ment Health.

[20] Laporte Uribe F, Gräske J, Grill S (2017). Regional dementia care networks in Germany: changes in caregiver burden at one-year follow-up and associated factors. Int Psychogeriatr.

[21] Zwingman I, Hoffmann W, Michalowsky B (2018). Supporting family dementia caregivers: testing the efficacy of dementia care management on multifaceted caregivers' burden. Aging Ment Health.

[22] Hajek A, Brettschneider C, Ernst A (2016). Longitudinal predictors of informal and formal caregiving time in community-dwelling dementia patients. Soc Psychiatry Psychiatr Epidemiol.

[23] Heinrich S, Laporte Uribe F, Wübbeler M (2016). Knowledge evaluation in dementia care networks: a mixed-methods analysis of knowledge evaluation strategies and the success of informing family caregivers about dementia support services. Int J Ment Health Syst.

[24] Geschke K, Scheurich A, Schermuly I, Laux N, Böttcher A, Fellgiebel A (2012). Effectivity of early psychosocial counselling for family caregivers in general practitioner based dementia care. Dtsch Med Wochenschr.

[25] Romero-Moreno R, Márquez-González M, Mausbach BT, Losada A (2012). Variables modulating depression in dementia caregivers: a longitudinal study. Int Psychogeriatr.

[26] Zwingmann I, Dreier-Wolfgramm A, Esser A (2020). Why do family dementia caregivers reject caregiver support services? Analyzing types of rejection and associated healthimpairments in a cluster-randomized controlled intervention trial. BMC Health Serv Res.

[27] Bulsara CE, Fynn N (2006). An exploratory study of gp awareness of carer emotional needs in Western Australia. BMC Fam Pract.

[28] Greenwood N, Mackenzie A, Habibi R, Atkins C, Jones R (2010). General practitioners and carers: a questionnaire survey of attitudes, awareness of issues, barriers and enablers to provision of services. BMC Fam Pract.

[29] Kassenärztliche Bundesvereinigung [National Association of Statutory Health Insurance Physicians]: Versichertenbefragung 2018. Ergebnisse einer repräsentativen Bevölkerungsumfrage [Results from a representative survey]. Berlin; 2018.

[30] Laux N, Melchinger H, Scheurich A (2010). Improving general practitioners guided dementia care. Dtsch Med Wochenschr.

[31] Gemeinsamer Bundesausschuss Innovationsausschuss [Federal Joint Committee, Innovation Committee]: DemStepCare – Hausarztbasierte Demenzversorgung mit koordinierter Kooperation und risikostratifiziertem Einsatz spezialisierter Pflegekräfte [Dementia care from general practitioners with coordinated cooperation and risk-ratified use of specialised care]. [https://innovationsfonds.g-ba.de/projekte/neue-versorgungsformen/demstepcare-hausarztbasierte-demenzversorgung-mit-koordinierter-kooperation-und-risikostratifiziertem-einsatz-spezialisierter-pflegekraefte.279] Accessed on 15 Sep 2021.

[32] Wangler J, Jansky M (2021). Support, needs and expectations of family caregivers regarding general practitioners - results from an online survey. BMC Fam Pract.

[33] Wangler J, Jansky M: What prevention potential does the general practitioner setting offer for family caregivers? – findings from a qualitative interview study. Wien Med Wochenschr 2021 (in press). 10.1007/s10354-021-00880-4 PMC10896783 34529149

[34] Wangler J, Jansky M: General practitioners‘ attitudes, procedures and challenges towards supporting family caregivers – results of a survey of primary care physicians. Dtsch med Wochenschr 2021 [Online ahead of print]. PMID:34794181. 10.1055/a-1671-8621. 10.1055/a-1671-8621 PMC8714302 34794181

[35] Wangler J, Fellgiebel A, Jansky M (2018). Dementia diagnosis in general practitioner care – attitudes, procedures and challenges from the perspective of general practitioners in Rhineland-Palatinate. Dtsch Med Wochenschr.

[36] Greenwood N, Mackenzie A, Harris R, Fenton W, Cloud G (2011). Perception of the role of general practice and practical support measures for carers of stroke survivors: a qualitative study. BMC Fam Pract.

[37] Höppner C, Schneemilch M, Lichte T (2015). Pflegende Angehörige und ihre Belastungen in Hausarztpraxen identifizieren – Hindernisse und Empfehlungen [Identifying Informal Carers and Their Burden in Family Practices – Barriers and Recommendations]. Z Allg Med.

[38] Mayring P (2010). Qualitative content analysis. Basics and Techniques.

[39] Burridge LH, Mitchell GK, Jiwa M, Girgis A (2011). Consultation etiquette in general practice: a qualitative study of what makes it different for lay cancer caregivers. BMC Fam Pract.

[40] Krug K, Bölter R, Ballhauen RA, Engeser P, Peters-Klimm F (2016). Burden experienced by family caregivers of patients at the end of life: what do general practice teams offer?. Gesundheitswesen.

[41] Lichte T, Beyer M, Mand P, et al.: Pflegende Angehörige – DEGAM-Leitlinie Nr.6 [Family caregivers – DEGAM guideline No. 6]. Available from: URL: http://www.degam.de/leitlinien-51.html. [Cited 2021 Sep 15].

[42] The princess Royal Trust for Carers and Royal College of general practitioners: supporting carers: an action guide for general practitioners and their teams. London: RCGP; 2011.

[43] Bruce DG, Glenys AP, Underwood PJ, Roberts D, Steed D (2002). Communication problems between general practitioners: effect on access to community support services. Med J Aust.

[44] Prüfer F, Joos S, Milksch A (2015). Die Rolle des Hausarztes in der kommunalen Gesundheitsförderung [the role of general practitioners in local health promotion]. Prävention und Gesundheitsförderung.

[45] Parmar J, Anderson S, Abbasi M (2021). Family Physician‘s and Primary Care Team's Perspectives on Supporting Family Caregivers in Primary Care Networks. Int J Environ Res Public Health.

[46] Carduff E, Finuance A, Kendall M (2014). Understanding the barriers to identifying carers of people with advanced illness in primary care: triangulating three data sources. BMC Fam Pract.

[47] Wangler J, Fellgiebel A, Jansky M (2019). The practice staff in primary care dementia recognition-is there an untapped potential?. Z Gerontol Geriat.

[48] Carlsen B, Bringedal B (2011). Attitudes to clinical guidelines – do GPs differ from other medical doctors?. BMJ Qual Saf.

[49] Carlsen B, Norheim OF (2008). "what lies beneath it all?" – an interview study of GPs' attitudes to the use of guidelines. BMC Health Serv Res.

[50] Cranney M, Warren E, Barton S (2001). Why do GPs not implement evidence-based guidelines? A descriptive study. Fam Pract.

[51] Plöthner M, Schmidt K, de Long L, Zeidler J, Damm K (2019). Needs and preferences of informal caregivers regarding outpatient care for the elderly: a systematic literature review. BMC Geriatr.

[52] Donath C, Gräßel E, Großfeld-Schmitz M (2010). Effects of general practitioner training and family support services on the care of home-dwelling dementia patients - results of a controlled cluster-randomized study. BMC Health Serv Res.

[53] Lane P, McKenna H, Ryan A (2003). The experience of the family caregivers’ role: a qualitative study. Res theory Nurs Pract.

[54] Pentzek M, Fuchs A, Abholz H-H, Wollny A (2011). Awareness of local dementia services among general practitioners with academic affiliation. Aging Clin Exp Res.

[55] Parmar J, Anderson S, Abbasi M (2020). Support for family caregivers: a scoping review of family physician’s perspectives on their role in supporting family caregivers. Health Soc Care Commun.

[56] Thyrian JR, Fiss T, Dreier A (2012). Life- and person-centred help in Mecklenburg-Western Pomerania, Germany (DelpHi): study protocol for a randomised controlled trial. Trials.

[57] Burridge LH, Mitchell G, Jiwa M (2017). Helping lay carers of people with advanced cancer and their GPs to talk: an exploration of Australian users’ views of a simple carer health checklist. Health Soc Care Commun.

[58] Kuske S, Graf R, Hartig M, Quasdorf T, Vollmar HC, Bartholomeyczik S (2014). Dementia considered? Safety-relevant communication between health care settings: a systematic review. J Public Health.

[59] Bablok I, Binder H, Graf E, Stelzer D (2021). Primary dementia care based on the individual needs of the patient: study protocol of the cluster randomised controlled study DemStepCare. BMC Geriatr.

[60] Hermann K, Boelter R, Engeser P (2012). PalliPA: How can general practices support caregivers of patients at their end of life in a home-care setting? A study protocol. BMC Res Notes.

[61] Mißlbeck A: Plädoyer für gemeinsame Behandlungspfade [A plea for common treatment pathways]. Available from: URL: https://www.aerztezeitung.de/Politik/Demenz-Plaedoyer-fuer-gemeinsame-Behandlungspfade-372175.html [Cited 2021 Sep 15].

[62] Radisch J, Baumgardt J, Touil E (2015). Dementia - treatment pathways for integrated outpatient care.

[63] Wolf D, Rhein C, Geschke K, Fellgiebel A (2019). Preventable hospitalizations among older patients with cognitive impairments and dementia. Int Psychogeriatr.

[64] Reilly S, Miranda-Castillo C, Malouf R (2015). Case management approaches to home support for people with dementia. Cochrane Database Syst Rev.

[65] Gibson C, Goeman D, Pond D (2020). What is the role of the practice nurse in the care of people living with dementia, or cognitive impairment, and their support person(s)?: a systematic review. BMC Fam Pract.

[66] Schoenmakers B, Buntinx F, Delepeleire J (2009). What is the role of the general practitioner towards the family caregiver of a community-dwelling demented relative? A systematic literature review. Scand J Prim Health Care.

[67] Grande G, Stajduhar K, Aoun S (2009). Supporting lay carers in end of life care: current gaps and future priorities. Palliat Med.

[68] Jack B, O’Brien M (2010). Dying at home: community nurses’ views on the impact of informal carers on cancer patients' place of death. Eur J Cancer Care (Engl).

[69] Thomas K (2003). Caring for the dying at home.

[70] Boyd KJ, Worth A, Kendall M (2009). Making sure services deliver for people with advanced heart failure: a longitudinal qualitative study of patients, family carers, and health professionals. Palliat Med.

[71] Griffiths J, Ewing G, Rogers M (2013). Early support visits by district nurses to cancer patients at home: a multi-perspective qualitative study. Palliat Med.

[72] Schneider N, Mitchell GK, Murray SA (2010). Palliative care in urgent need of recognition and development in general practice: the example of Germany. BMC Fam Pract.

